# The relationship between pain and disruptive behaviors in nursing home resident with dementia

**DOI:** 10.1186/1471-2318-13-14

**Published:** 2013-02-11

**Authors:** Hyochol Ahn, Ann Horgas

**Affiliations:** 1 Department of Adult and Elderly Nursing, College of Nursing, University of Florida, Gainesville, FL, 32610-0197, USA

**Keywords:** Disruptive behaviors, Pain, Dementia, Nursing home

## Abstract

**Background:**

Nursing home residents with dementia gradually lose the ability to process information so that they are less likely to express pain in typical ways. These residents may express pain through disruptive behaviors because they cannot appropriately verbalize their pain experience. The objective of this study was to investigate the effect of pain on disruptive behaviors in nursing home residents with dementia.

**Methods:**

This is a secondary analysis of the Minimum Data Set (MDS 2.0) assessment data on long-term care from the state of Florida. The data used in this study were the first comprehensive assessment data from NH residents with dementia aged 65 and older (N = 56,577) in Medicare- or Medicaid-certified nursing homes between January 1, 2009 and December 31, 2009. Variables examined were pain, wandering, aggression, agitation, cognitive impairment, activities of daily living impairments, and demographic characteristics. Ordinal logistic regression was used to evaluate the effect of pain on disruptive behaviors.

**Results:**

Residents with more severe pain are less likely to display wandering behaviors (OR = .77, 95% CI for OR = [0.73, 0.81]), but more likely to display aggressive and agitated behaviors (OR = 1.04, 95% CI for OR = [1.01, 1.08]; OR = 1.17, 95% CI for OR = [1.13, 1.20]).

**Conclusions:**

The relationship between pain and disruptive behaviors depends on the type of behaviors. Pain is positively correlated with disruptive behaviors that do not involve locomotion (e.g., aggression and agitation), but negatively related to disruptive behaviors that are accompanied by locomotion (e.g., wandering). These findings indicate that effective pain management may help to reduce aggression and agitation, and to promote mobility in persons with dementia.

## Background

Pain assessment in nursing home (NH) residents with dementia is challenging due to cognitive and communicative impairments. Pain self-report, the gold standard assessment in cognitively intact persons, is questionable in cognitively impaired NH residents because dementia impairs their ability to remember, interpret, and respond to pain [1,2]. NH residents with dementia gradually lose the ability to process information so that they are less likely to express pain in typical ways, even when there is a probable cause for pain [1]. Therefore, pain is often under-reported in NH residents with dementia. These residents may express pain through disruptive behaviors [3], because they cannot appropriately verbalize their pain experience.

Disruptive behaviors, also known as “problematic behaviors,” “disturbing behaviors,” or “challenging behaviors,” refer to inappropriate, repetitive, or dangerous behaviors that are disruptive to the living and working environment in the NH [4,5]. Among many disruptive behaviors, three behaviors are most prominent in the current literature: wandering behaviors, aggressive behaviors, and agitated behaviors [6,7]. Wandering occurs in approximately 40 to 60% of NH residents with dementia [8], and aggression and agitation occurs in about 50% to 80% of NH residents with cognitive impairments [9].

Disruptive behaviors are problematic to NH residents and staff. Disruptive behaviors are associated with injuries and hospitalizations among NH residents with dementia, and contribute to stress and burnout among caregivers [10,11]. The cost of care for NH residents with dementia is three times higher than that of other NH residents, and about 30% of these costs are attributed to the management of disruptive behaviors [12]. Psychoactive medications or restraints are often used to manage disruptive behaviors [13]; however, these often lead to falls, impaired functioning, and decreased mobility. The use of restraints is also an affront to personal dignity. The better approach to managing disruptive behaviors is to control their possible causes, such as pain.

Thus, the purpose of this study is to explore the relationship between pain and disruptive behaviors in NH residents with dementia. Such information may identify potential new intervention approaches for managing these behaviors.

### Theoretical framework

**Figure 1:**
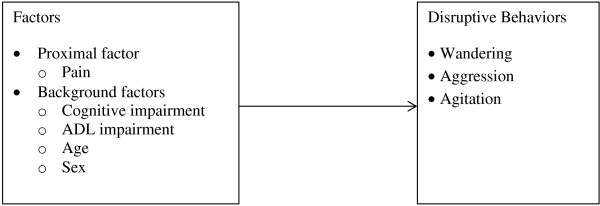
Theoretical framework adapted from the Need-driven Dementia-compromised Behavior (NDB) model.

The Need-driven Dementia-compromised Behavior (NDB) model [14] was used to guide this study of the relationship between pain and disruptive behaviors in NH residents with dementia (Figure 1). The NDB model posits two main constructs that are associated with dementia-compromised behaviors: background factors and proximal factors. Background factors represent those characteristics that place older adults at risk for disruptive behaviors. Proximal factors represent the conditions under which disruptive behaviors occur. We conceptualized pain as a proximal factor that would have a direct relationship with disruptive behaviors (e.g., wandering, aggression, and agitation). For this study, the level of cognitive impairment, activities of daily living (ADL) impairment, and demographic characteristics (e.g., age and sex) represent background factors. These variables were selected as covariates because they have established relationships with both pain and disruptive behaviors [15-18] and may influence the relationship between pain and disruptive behaviors.

## Methods

This is a secondary analysis of the nursing home Minimum Data Set (MDS) from the state of Florida during calendar year 2009. The first comprehensive assessment for each NH resident was used in this study. The archived data files of the most recent version of MDS (MDS 3.0) were not yet available to researchers, but are due for release in early 2013 [19]. The MDS data are mandatory in all NHs certified to participate in Medicare and Medicaid. Approval for the study was obtained from the University of Florida Health Science Center Institutional Review Board.

The MDS assessment data, standardized data on residents’ status based on routine and continuous observations by nursing staff, provides comprehensive information on all the NH residents. The MDS assessment is completed on admission to the facility, on a quarterly basis thereafter, and upon significant changes in status [20]. The complete federal database consists of over 1.5 million older adults who live in NHs throughout the United States. Although it is used primarily for clinical purposes, the MDS has also been used for research on cognition and behavioral symptoms in this population [21-23]. Several MDS subscales have been created and evaluated, and have demonstrated acceptable reliability and validity: MDS-Pain severity scale [24], MDS-Depression Rating Scale [25], MDS-Aggression Behavior Scale [26], MDS-Challenging Behavior Profile [27], MDS-Discomfort Behavior Scale [28], MDS-Cognitive Performance Scale [28,29], MDS-index of social engagement [30,31], MDS-Activities of Daily Living scale [32,33], Resident Assessment Instrument-Mental Health [34], and MDS-Change in Health, End-stage disease and Signs and Symptoms [35]. Details of the reliability and validity coefficients for each of the major study variables are described in the measurement section.

### Data used in this study

The data used in this study were collected on residents with dementia in Medicare- or Medicaid-certified NHs who have a MDS comprehensive assessment on file. The data were acquired from the Centers for Medicare & Medicaid Services. Selection criteria were applied to ascertain data from NH residents older than 65 years old with Alzheimer’ disease or other dementia, based on documented medical diagnosis. Data from comatose residents were excluded, because these residents cannot display the disruptive behaviors investigated in this study. This selection process yielded 56,577 unique cases for the analyses.

The sample is mostly female (67.7%), and a mean age of 84 (years range = 65–109). The prevalence of disruptive behaviors is as follows: wandering behaviors (9.0%), aggressive behaviors (24.4%), and agitated behaviors (24.1%) (Table 1).

**Table 1:** Sample characteristics

**Characteristic**	**Number**	**Total sample**
Age, mean ± SD	56577	84.37 ± 7.43
Gender, n (%)	56566	
Male		18,265 (32.3)
Female		38,301 (67.7)
MDS-CPS, mean ± SD	56543	3.17 ± 1.52
MDS-ADL, mean ± SD	56577	18.66 ± 6.41
Pain severity, mean ± SD	56568	0.48 ± 0.70
Wandering behaviors, n (%)	56573	
No wandering (MDS-wandering = 0)		51,463 (91.0)
1-3 days in 7 days (MDS-wandering = 1)		2,637 (4.7)
4-6 days in 7 days (MDS-wandering = 2)		994 (1.8)
Wandering daily (MDS-wandering = 3)		1,479 (2.6)
Aggressive behaviors, n (%)	56572	
None (MDS-ABS = 0)		42,764 (75.6)
Moderate (MDS-ABS = 1 – 2)		9,667 (17.1)
Severe (MDS-ABS = 3 – 5)		3,390 (6.0)
Very severe (MDS-ABS = 6 – 12)		751 (1.3)
Agitated behaviors, n (%)	56571	
None (revised MDS-CBP agitation = 0)		42,941 (75.9)
Mild (revised MDS-CBP agitation = 1)		6,916 (12.2)
Moderate (revised MDS-CBP agitation = 2)		5,099 (9.0)
Severe (revised MDS-CBP agitation = 3)		1,615 (2.9)

*MDS-CPS* = MDS-Cognitive Performance Scale.

*MDS-ADL* = MDS-Activities of Daily Living impairment scale.

*MDS-ABS* = MDS-Aggression Behavior Scale.

*Revised MDS-CBP agitation* = revised MDS-Challenging Behavior Profile, agitation subscale.

### Measurement

MDS subscales and items were used to indicate the main study concepts: pain, wandering, aggression, and agitation. These are described below.

#### Pain

The MDS-pain severity scale [24], combining both pain frequency (0 = no pain, 1 = pain less than daily, and 2 = pain daily) and pain intensity (1 = mild pain, 2 = moderate pain, and 3 = horrible or excruciating pain), was used to assess pain severity in NH residents with dementia. This scale can range from 0 to 3, with higher scores indicating greater pain severity. NH residents’ self-report is reflected in the MDS pain items if residents can self-report and staff completing the MDS assessments have confidence in residents’ self-report. Otherwise, the staff who complete the MDS assessment document pain symptoms based on proxy reports from facility nursing staff that provides care to the residents. The MDS-pain severity scale has been reported to have an inter-rater reliability coefficient of 0.73, and kappa coefficient of 0.70 with a Visual Analogue Scale in a study involving 95 U.S. nursing home residents at 25 Medicare-certified skilled nursing facilities in Massachusetts [24].

#### Disruptive behaviors

The MDS-wandering item was used to measure the frequency of wandering in the last 7 days. Wandering frequency is recorded by staff observation. It is recorded as no wandering, wandering occurred 1 to 3 days, wandering occurred 4 to 6 days, and daily wandering. The wandering item has been reported to have a reliability coefficient of 0.63, and an inter-rater reliability of 0.95 [36,37].

The MDS-Aggression Behavior Scale (MDS-ABS) was used to measure the frequency of aggressive behaviors. The MDS-ABS is a sum score of four MDS items: verbally abusive behavioral symptoms, physically abusive behavioral symptoms, socially inappropriate behavioral symptom, and resisting care. The MDS-ABS can range from 0 to 12, with higher scores indicating more frequent aggressive behaviors. The MDS-ABS has been reported to have an internal consistency reliability of 0.79 to 0.95, and a criterion validity coefficient of 0.72 with Cohen-Mansfield Agitation Inventory aggression subscale scores [26].

The revised MDS-Challenging Behavior Profile (MDS-CBP) agitation subscale was used to assess the frequency of agitated behaviors. The revised agitation scores, calculated using two MDS items (e.g., periods of restlessness and repetitive physical movements), can range from 0 to 3, with higher scores indicating more frequent agitated behaviors. This revised agitation scale has Cronbach’s alpha coefficient of .68. The original MDS-CBP agitation subscale, computing from 4 MDS items (e.g., periods of restlessness, repetitive physical movements, wandering, and socially inappropriate behavioral symptom), has been reported to have Cronbach’s alpha coefficient of 0.70, inter-rater reliability of 0.61, and a Spearman’s rank correlation coefficient of 0.50 with Behavior Rating Scale for Psychogeriatric Inpatients [27].

#### Background factors

The MDS-cognitive performance scale (MDS-CPS) [38] was used to measure the level of cognitive impairment. The MDS-CPS score is calculated using five MDS items: comatose, short-term memory, cognitive skills or daily decision making, making oneself understood, and self-performance in eating. The MDS-CPS can range from 0 to 6, with higher scores indicating more cognitive impairment. The MDS-CPS has been reported to have a kappa coefficient of 0.45-0.75 with Mini-Mental State Examination, a kappa coefficient of 0.41-0.77 against Global Deterioration Scale, a kappa coefficient of 0.66 against Psychogeriatric Dependency Rating Scale, a kappa coefficient of 0.45 against Mattis Dementia Rating Scale [29,38-41].

The MDS-Activities of Daily Living-Long Form (MDS ADL-Long Form) [42] was used to measure the level of ADL impairment. The MDS ADL-Long Form scores are calculated using 7 MDS items: self-performance of bed mobility, transfer, locomotion on unit, dressing, eating, toilet use, and personal hygiene. MDS ADL-Long Form can range from 0 to 28, with higher scores indicating more impairment of ADLs. The MDS ADL-Long Form has been reported to have a reliability coefficient of 0.92-0.97, an inter-rater reliability coefficient of 0.61-0.95, and a kappa coefficient of 0.58 – 0.79 against Physical Self-Maintenance Scale [31,43].

Demographics characteristics (e.g., age and gender) were collected from the MDS form. Age was a continuous variable and gender was dichotomous (0 = female; 1 = male). They were included as covariates in the analyses.

### Statistical analysis

Analyses were performed using SPSS, version 20 (IBM Inc., Armonk, NY). Multivariate analyses were conducted to explore the relationship between pain and disruptive behaviors in this sample. Aggression was severely positively skewed, and none of the transformations (e.g., logarithmic transformation, square root transformation, inverse transformation, and square transformation) resolved the normal distribution issue. Therefore, aggression was collapsed into four groups (none, moderate, severe, and very severe), based on published algorithms in the literature [26]. Aggression was transformed as none (MDS-ABS = 0), moderate (MDS-ABS = 1–2), severe (MDS-ABS = 3–5), and very severe (MDS-ABS = 6–12). Due to concerns that NH residents who take psychotropic medications (e.g., antipsychotics, antidepressants, etc.) may exhibit less frequent disruptive behaviors [44], we re-ran the statistical analysis excluding these subjects.

Since the level of measurement of the dependent variables was ordinal, logistic regression for ordinal variables was used to evaluate the effect of pain severity on the three disruptive behaviors, after controlling for covariates. Using the same independent variables in analysis with different dependent variables carries the risk of inflating the Type I error. To keep the overall risk of a Type I error to the 5% level, p-value for the each regression analysis is set at .017.

## Results

The results of ordinal logistic regression on three disruptive behaviors, after controlling for covariates (e.g., the level of cognitive impairment, the level of ADL impairment, and sociodemographic factors) are described below.

### The effect of pain on wandering behaviors

Pain severity is negatively associated with the frequency of wandering behaviors (Table 2). NH residents with more severe pain are less likely to display wandering behaviors (Logistic regression coefficient = −0.26, p < .001, Odds Ratio = .77, 95% CI for Odds Ratio = [0.73, 0.81]).

**Table 2:** Predicting disruptive behaviors from pain severity, after controlling for covariates (N = 56,577)

**Variables**	**Wandering**			**Aggression**			**Agitation**		
	**B**	**OR**	**95% CI for OR**	**B**	**OR**	**95% CI for OR**	**B**	**OR**	**95% CI for OR**
**Independent Variable**									
Pain	−0.26^*^	0.77	[0.73, 0.81]	0.04^*^	1.04	[1.01, 1.08]	0.15^*^	1.17	[1.13, 1.20]
**Covariates**									
MDS-CPS	0.68^*^	1.97	[1.91, 2.02]	0.36^*^	1.43	[1.41, 1.46]	0.46^*^	1.58	[1.55, 1.60]
MDS-ADL	−0.15^*^	0.87	[0.86, 0.87]	−0.03^*^	0.98	[0.97, 0.98]	−0.02^*^	0.98	[0.97, 0.98]
Age	−0.01^*^	0.99	[0.98, 0.99]	−0.01^*^	0.99	[0.99, 0.99]	−0.01^*^	0.99	[0.99, 0.99]
Sex									
*Male*	0.22^*^	1.25	[1.17, 1.33]	0.28^*^	1.33	[1.27, 1.39]	0.24^*^	1.27	[1.22, 1.33]
*Female*	0.00	1.00		0.00	1.00			1.00	

Nagelkerke R-square: Wandering = 0.15, Aggression = 0.06, Agitation = 0.08. B = logistic regression coefficient, OR = Odds Ratio = Exp(B). MDS-CPS = MDS-Cognitive Performance Scale. MDS-ADL = MDS-Activities of Daily Living impairment scale. ^*^p < .001.

### The effect of pain on aggressive behaviors

Pain severity is positively associated with the frequency of aggressive behaviors (Table 2). NH residents with more severe pain are more likely to display aggressive behaviors (Logistic regression coefficient = 0.04, p < .001, Odds Ratio = 1.04, 95% CI for Odds Ratio = [1.01, 1.08]).

### The effect of pain on agitated behaviors

Pain severity is positively associated with the frequency of agitated behaviors (Table 2). NH residents with more severe pain are more likely to display agitated behaviors (Logistic regression coefficient = 0.15, p < .001, Odds Ratio = 1.17, 95% CI for Odds Ratio = [1.13, 1.20]).

### The study results in subsample without psychotropic medications

The results of ordinal logistic regression in the subsample without psychotropic medications (e.g., antipsychotics, antidepressants, etc.) are summarized in Table 3. These results of ordinal logistic regression are similar when NH residents who used psychotropic medications in the past 7 days were excluded. Pain severity is negatively associated with the frequency of wandering behaviors, but positively associated with the frequency of aggressive and agitated behaviors.

**Table 3:** The study results in subsample without psychotropic medications (N = 17,435)

**Variables**	**Wandering**			**Aggression**			**Agitation**		
	**B**	**OR**	**95% CI for OR**	**B**	**OR**	**95% CI for OR**	**B**	**OR**	**95% CI for OR**
**Independent Variable**									
Pain	−0.33^*^	0.72	[0.63, 0.83]	0.07^**^	1.07	[1.01, 1.15]	0.15^*^	1.16	[1.08, 1.25]
**Covariates**									
MDS-CPS	0.63^*^	1.87	[1.76, 2.00]	0.29^*^	1.34	[1.29, 1.38]	0.42^*^	1.53	[1.47, 1.58]
MDS-ADL	−0.15^*^	0.86	[0.85, 0.87]	−0.04^*^	0.97	[0.96, 0.97]	−0.04^*^	0.96	[0.96, 0.97]
Age	0.00	1.00	[0.99, 1.01]	0.01^*^	1.01	[1.00, 1.02]	0.00	1.00	[1.00, 1.01]
Sex									
*Male*	0.12	1.12	[0.96, 1.31]	0.19^*^	1.21	[1.10, 1.33]	0.22^*^	1.24	[1.13, 1.37]
*Female*	0.00	1.00		0.00	1.00			1.00	

Nagelkerke R-square: Wandering = 0.13, Aggression = 0.03, Agitation = 0.05. B = logistic regression coefficient, OR = Odds Ratio = Exp(B). MDS-CPS = MDS-Cognitive Performance Scale. MDS-ADL = MDS-Activities of Daily Living impairment scale. ^*^p < .001. ^**^p < .05.

## Discussion

It was found that more severe pain is associated with less frequent wandering behaviors, but more frequent aggressive and agitated behaviors, after controlling for covariates. Most of the published literature suggested that there is a positive relationship between pain and disruptive behaviors in general [6,11,45]. However, the results of this study suggest that the relationship between pain and disruptive behaviors depends on the type of behaviors examined. The direction of the relationship between these variables depends on whether the disruptive behaviors are accompanied by locomotion. Pain is positively correlated with disruptive behaviors that do not involve locomotion (e.g., aggression and agitation), but negatively related to disruptive behaviors that are accompanied by locomotion (e.g., wandering). That is, residents who experience more severe pain are more likely to display aggression and agitation, and less likely to move around.

The finding that pain and aggressive or agitated behaviors are positively linked in NH residents with dementia is consistent with other published reports. Buffum and colleagues [46] reported that pain was positively related to agitation (*r* = .50, *p* = .003) using a bivariate correlation analysis in 33 Veterans Affairs NH residents with dementia. Manfredi and colleagues [47] demonstrated that opioid treatment for pain reduced agitation in 13 NH residents with dementia who were more than 85 years old (mean change in CMAI score: -6.4, 95% CI [−10.96, -1.8]). Both of these studies have a small sample size. Thus, the results of this study using a large sample from all the nursing home residents with dementia in the state of Florida substantiates and extends the positive relationship between pain and non-locomotive disruptive behaviors from these previous findings.

In contrast, the finding on the relationship between pain and wandering behavior in this study is opposite to the findings presented in the literature review. Kiely and colleagues [48] used MDS assessment data from 8,982 NH residents, and reported that NH residents who expressed sadness or pain in MDS assessment data were 65% more likely to develop wandering behaviors than their counterparts who did not express sadness or pain (OR = 1.65, p = .02). Our study measured pain more specifically using the MDS-pain severity scale [24], combining both pain frequency and pain intensity, while Kiely and the colleagues [48] measured pain by a dichotomized expression of sadness or pain. Sadness is not typically considered an indicator of pain, and its inclusion may have confounded pain and depression or mood disorder.

Several limitations of this study should be noted. First, this study is inherently limited by secondary analysis of federally mandated MDS assessment data, and the effect of clustering within facility is not controlled in this study. The variables and the procedures cannot be controlled. The MDS assessment data may have some variability due to different styles and skills of MDS coordinators in each facility. Second, the role of pain medications is not considered in this study. The highest level of pain could have been managed by pain medications, but it is not possible to discern this in the MDS assessment data. However, similar to our study, most of the literature reported the relationship between highest level of pain and the frequency of behavioral symptoms during the observation period without controlling for pain medications [11,49]. Third, the amount of variance in disruptive behaviors that is explained by these logistic regression models is small (ranging from 6% to 15%). This suggests that there are other factors that contribute to disruptive behaviors that were not specified in our models. Finally, this study design is descriptive and cross-sectional. As such, this study is not able to examine causal relationships between pain and disruptive behaviors.

Findings from this study can be a foundation for future research. Studies using prospective designs are needed to validate these findings. Also, randomized controlled trials can be used to compare comprehensive pain management and usual pain management with regard to the frequency of disruptive behaviors. This type of study can provide evidence for causal relationships between pain management and disruptive behaviors and support changes in clinical practice. Third, future research would include the longitudinal MDS assessment data to examine trends over time. The longitudinal nature of MDS assessment data, collected every three months or more often, provides an opportunity to describe change over time, and facilitates the use of more powerful statistical analysis techniques to describe both within- and between-person changes.

## Conclusions

Pain exacerbated disruptive behaviors that are not locomotion-based. In order to reduce these disruptive behaviors, their underlying causes, such as pain, should be investigated and well managed. However, pain assessment in cognitively impaired residents can be challenging. Comprehensive pain assessment should be developed further, and pain should be well controlled to reduce these problematic disruptive behaviors.

## Abbreviations

ADL: Activities of daily living; MDS: Minimum data set; MDS-ABS: MDS-Aggression behavior scale; MDS-CBP: MDS-Challenging behavior profile; MDS-CPS: MDS-Cognitive performance scale; NH: Nursing home.

## Competing interests

The authors have no financial or any other kind of personal conflicts with this manuscript. This study was supported by Grant award from Sigma Theta Tau Alpha Theta Chapter.

## Authors’ contributions

HA conceptualized the study, completed all statistical analyses, and wrote the manuscript. AH provided oversight and consultation during all aspects of the study. Both authors read and approved the final manuscript.

## Pre-publication history

The pre-publication history for this paper can be accessed here:

http://www.biomedcentral.com/1471-2318/13/14/prepub

## References

[B1] Horgas A Elliott AF Marsiske M Pain assessment in persons with dementia: Relationship between self-report and behavioral observation J Am Geriatr Soc 2009 57 1 126 132 10.1111/j.1532-5415.2008.02071.x 19054191 PMC2712941

[B2] Cohen-Mansfield J The adequacy of the minimum data set assessment of pain in cognitively impaired nursing home residents J Pain Symptom Manage 2004 27 4 343 351 10.1016/j.jpainsymman.2004.01.001 15050662

[B3] Burfield AH Wan TT Sole ML Cooper JW Behavioral cues to expand a pain model of the cognitively impaired elderly in long-term care Clin Interv Aging 2012 7 207 223 22807630 10.2147/CIA.S29656 PMC3396050

[B4] Kovach CR Noonan PE Schlidt AM Reynolds S Wells T The serial trial intervention: an innovative approach to meeting needs of individuals with dementia J Gerontol Nurs 2006 32 4 18 25 16615709 10.3928/00989134-20060401-05

[B5] Pieper MJ Achterberg WP Francke AL van der Steen JT Scherder EJ Kovach CR The implementation of the serial trial intervention for pain and challenging behaviour in advanced dementia patients (STA OP!): a clustered randomized controlled trial BMC Geriatr 2011 11 1 12 10.1186/1471-2318-11-12 21435251 PMC3072328

[B6] Aalten P van Valen E de Vugt ME Lousberg R Jolles J Verhey FR Awareness and behavioral problems in dementia patients: a prospective study Int Psychogeriatr 2006 18 1 3 17 10.1017/S1041610205002772 16388704

[B7] Aud MA Dangerous wandering: elopements of older adults with dementia from long-term care facilities Am J Alzheimers Dis Other Demen 2004 19 6 361 368 10.1177/153331750401900602 15633945 PMC10833955

[B8] Sink KM Covinsky KE Newcomer R Yaffe K Ethnic differences in the prevalence and pattern of dementia-related behaviors J Am Geriatr Soc 2004 52 8 1277 1283 10.1111/j.1532-5415.2004.52356.x 15271114

[B9] Kunik ME Walgama JP Snow AL Davila JA Schulz PE Steele AB Morgan RO Documentation, assessment, and treatment of aggression in patients with newly diagnosed dementia Alzheimer Dis Assoc Disord 2007 21 2 115 121 10.1097/WAD.0b013e318065c4ba 17545736

[B10] Kunik ME Snow AL Davila JA McNeese T Steele AB Balasubramanyam V Doody R Schulz PE Kalavar JS Walder A Morgan RO Consequences of aggressive behavior in patients with dementia J Neuropsychiatry Clin Neurosci 2010 22 1 40 47 10.1176/appi.neuropsych.22.1.40 20160208

[B11] Norton MJ Allen RS Snow AL Hardin JM Burgio LD Predictors of need-driven behaviors in nursing home residents with dementia and associated certified nursing assistant burden Aging Ment Health 2010 14 3 303 309 10.1080/13607860903167879 20425649

[B12] 2011 Alzheimer’s Disease facts and figures http://www.alz.org/downloads/Facts_Figures_2011.pdf

[B13] Hersch EC Falzgraf S Management of the behavioral and psychological symptoms of dementia Clin Interv Aging 2007 2 4 611 621 18225462 10.2147/cia.s1698 PMC2686333

[B14] Algase DL Beck C Kolanowski A Whall A Berent S Richards K Beattie E Need-driven dementia-compromised behavior: An alternative view of disruptive behavior Am J Alzheimers Dis Other Demen 1996 11 6 10 19 10.1177/153331759601100603

[B15] Burgio LD Park NS Hardin JM Sun F A longitudinal examination of agitation and resident characteristics in the nursing home Gerontologist 2007 47 5 642 649 10.1093/geront/47.5.642 17989406

[B16] Cohen-Mansfield J Libin A Verbal and physical non-aggressive agitated behaviors in elderly persons with dementia: Robustness of syndromes J Psychiatr Res 2005 39 3 325 332 10.1016/j.jpsychires.2004.08.009 15725431

[B17] Reynolds KS Hanson LC DeVellis RF Henderson M Steinhauser KE Disparities in pain management between cognitively intact and cognitively impaired nursing home residents J Pain Symptom Manage 2008 35 4 388 396 10.1016/j.jpainsymman.2008.01.001 18280101

[B18] Shega JW Ersek M Herr K Paice JA Rockwood K Weiner DK Dale W The multidimensional experience of noncancer pain: Does cognitive status matter? Pain Medicine 2010 11 11 1680 1687 10.1111/j.1526-4637.2010.00987.x 21044258

[B19] Long Term Care Minimum Data Set 3.0 http://www.resdac.org/cms-data/files/mds-3.0

[B20] Lum TY Lin W-C Kane RL Use of proxy respondents and accuracy of minimum data set assessments of activities of daily living J Gerontol A Biol Sci Med Sci 2005 60 5 654 659 10.1093/gerona/60.5.654 15972620

[B21] Won A Lapane KL Vallow S Schein J Morris JN Lipsitz LA Long-term effects of analgesics in a population of elderly nursing home residents with persistent nonmalignant pain J Gerontol A Biol Sci Med Sci 2006 61 2 165 169 10.1093/gerona/61.2.165 16510860 PMC2276585

[B22] Lee FP Leppa C Schepp K Using the minimum data set to determine predictors of terminal restlessness among nursing home residents J Nurs Res 2006 14 4 286 296 10.1097/01.JNR.0000387588.12340.d1 17345758

[B23] Carpenter GI Hastie CL Morris JN Fries BE Ankri J Measuring change in activities of daily living in nursing home residents with moderate to severe cognitive impairment BMC Geriatr 2006 6 1 8 10.1186/1471-2318-6-1 16584565 PMC1522014

[B24] Fries BE Simon SE Morris JN Flodstrom C Flodstrom C Bookstein FL Pain in U.S. Nursing homes: validating a pain scale for the minimum data set Gerontologist 2001 41 2 173 179 10.1093/geront/41.2.173 11327482

[B25] Burrows AB Morris JN Simon SE Hirdes JP Phillips C Development of an minimum data set-based depression rating scale for use in nursing homes Age Ageing 2000 29 165 172 10.1093/ageing/29.2.165 10791452

[B26] Perlman CM Hirdes JP The aggressive behavior scale: a new scale to measure aggression based on the minimum data Set J Am Geriatr Soc 2008 56 12 2298 2303 10.1111/j.1532-5415.2008.02048.x 19093929

[B27] Gerritsen DL Achterberg WP Steverink N Pot AM Frijters DH Ribbe MW The MDS challenging behavior profile for long-term care Aging Ment Health 2008 12 1 116 123 10.1080/13607860701529882 18297486

[B28] Stevenson KM Brown RL Dahl JL Ward SE Brown MS The discomfort behavior scale: a measure of discomfort in the cognitively impaired based on the minimum data Set 2.0 Res Nurs Health 2006 29 6 576 587 10.1002/nur.20168 17131282

[B29] Hartmaier SL Sloane PD Guess HA Koch GG The MDS cognition scale: a valid instrument for identifying and staging nursing home residents with dementia using the minimum data set J Am Geriatr Soc 1994 42 11 1212 1213 7963204 10.1111/j.1532-5415.1994.tb06984.x

[B30] Achterberg W Pot AM Kerkstra A Ooms M Muller M Ribbe M The effect of depression on social engagement in newly admitted Dutch nursing home residents Gerontologist 2003 43 2 213 218 10.1093/geront/43.2.213 12677078

[B31] Sgadari A Morris JN Fries BE Ljunggren G Jonsson PV DuPaquier JN Schroll M Efforts to establish the reliability of the resident assessment instrument Age Ageing 1997 26 Suppl 2 27 30 10.1093/ageing/26.suppl_2.27 9464551

[B32] Williams BC Li Y Fries BE Warren RL Predicting patient scores between the functional independence measure and the minimum data set: Development and performance of a FIM-MDS “crosswalk” Arch Phys Med Rehabil 1997 78 1 48 54 10.1016/S0003-9993(97)90009-5 9014957

[B33] Frederiksen K Tariot P De Jonghe E Minimum Data Set Plus (MDS+) scores compared with scores from five rating scales J Am Geriatr Soc 1996 44 3 305 309 8600202 10.1111/j.1532-5415.1996.tb00920.x

[B34] Hirdes JP Smith TF Rabinowitz T Yamauchi K Perez E Telegdi NC Prendergast P Morris JN Ikegami N Phillips CD Fries BE The resident assessment instrument-mental health (RAI-MH): inter-rater reliability and convergent validity J Behav Health Serv Res 2002 29 4 419 432 10.1007/BF02287348 12404936

[B35] Hirdes JP Frijters DH Teare GF The MDS-CHESS scale: a new measure to predict mortality in institutionalized older people J Am Geriatr Soc 2003 51 1 96 100 10.1034/j.1601-5215.2002.51017.x 12534853

[B36] Casten R Lawton MP Parmelee PA Kleban MH Psychometric characteristics of the minimum data set I: confirmatory factor analysis J Am Geriatr Soc 1998 46 6 726 735 9625189 10.1111/j.1532-5415.1998.tb03808.x

[B37] Lawton MP Casten R Parmelee PA Haitsma KV Corn J Kleban MH Psychometric characteristics of the minimum data Set II: validity J Am Geriatr Soc 1998 46 6 736 744 9625190 10.1111/j.1532-5415.1998.tb03809.x

[B38] Morris JN Fries BE Mehr DR Hawes C Phillips C Mor V Lipsitz LA MDS cognitive performance scale J Gerontol A Biol Sci Med Sci 1994 49 4 174 182 10.1093/geronj/49.4.m174 8014392

[B39] Bula CJ Wietlisbach V Use of the cognitive performance scale (CPS) to detect cognitive impairment in the acute care setting: Concurrent and predictive validity Brain Res Bull 2009 80 4–5 173 178 19559765 10.1016/j.brainresbull.2009.05.023

[B40] Hartmaier SL Sloane PD Guess HA Koch GG Mitchell CM Phillips CD Validation of the minimum data Set cognitive performance scale: agreement with the mini-mental state examination J Gerontol A Biol Sci Med Sci 1995 50A 2 128 133 10.1093/gerona/50A.2.M128 7874589

[B41] McConnell ES Pieper CF Sloane RJ Branch LG Effects of cognitive performance on change in physical function in long-stay nursing home residents J Gerontol A Biol Sci Med Sci 2002 57 12 778 784 10.1093/gerona/57.12.M778 12456736

[B42] Morris JN Fries BE Morris SA Scaling ADLs within the MDS J Gerontol A Biol Sci Med Sci 1999 54 11 546 553 10.1093/gerona/54.11.M546 10619316

[B43] Hawes C Morris JN Phillips CD Mor V Fries BE Nonemaker S Reliability estimates for the Minimum Data Set for nursing home resident assessment and care screening (MDS) Gerontologist 1995 35 2 172 178 10.1093/geront/35.2.172 7750773

[B44] Pollock BG Mulsant BH Rosen J Sweet RA Mazumdar S Bharucha A Marin R Jacob NJ Huber KA Kastango KB Chew ML Comparison of citalopram, perphenazine, and placebo for the acute treatment of psychosis and behavioral disturbances in hospitalized, demented patients Am J Psychiatry 2002 159 3 460 465 10.1176/appi.ajp.159.3.460 11870012

[B45] Villanueva MR Smith TL Erickson JS Lee AC Singer CM Pain Assessment for the Dementing Elderly (PADE): Reliability and validity of a new measure J Am Med Dir Assoc 2003 4 1 1 8 10.1016/S1525-8610(04)70257-1 12807590

[B46] Buffum MD Miaskowski C Sands L Brod M A pilot study of the relationship between discomfort and agitation in patients with dementia Geriatr Nur (Lond) 2001 22 2 80 85 10.1067/mgn.2001.115196 11326214

[B47] Manfredi PL Breuer B Wallenstein S Stegmann M Bottomley G Libow L Opioid treatment for agitation in patients with advanced dementia Int J Geriatr Psychiatry 2003 18 8 700 705 10.1002/gps.906 12891637

[B48] Kiely DK Morris JN Algase DL Resident characteristics associated with wandering in nursing homes Int J Geriatr Psychiatry 2000 15 11 1013 1020 10.1002/1099-1166(200011)15:11<1013::AID-GPS226>3.0.CO;2-X 11113981

[B49] Bartels SJ Horn SD Smout RJ Dums AR Flaherty E Jones JK Monane M Taler GA Voss AC Agitation and depression in frail nursing home elderly patients with dementia: treatment characteristics and service use Am J Geriatr Psychiatry 2003 11 2 231 238 12611753

